# Assessing influenza-related mortality: Comment on Zucs *et al.*

**DOI:** 10.1186/1742-7622-2-7

**Published:** 2005-07-21

**Authors:** Jonathan Dushoff

**Affiliations:** 1 Princeton U. Dept. of Ecology and Evolutionary Biology, Princeton NJ, 08544, USA; 2 National Institutes of Health, Fogarty International Center, Bethesda MD, 20892, USA

## 

Influenza is an important source of mortality and morbidity, and an important public health priority. Measuring the health burden imposed by influenza viruses is an important, and still controversial, question. Some authors argue that influenza is directly or indirectly responsible for the majority of seasonal excess deaths in temperate countries [1], while others argue that they trigger only a small minority [2]. Retrospective cohort studies have shown a surprisingly large protective effect of influenza vaccination against deaths from any cause [3-5], and one author has provocatively suggested that increased influenza vaccination of the elderly could halve the total mortality rate [6]. The population-level interpretation of these cohort studies is not clear, however [7], and studies in the Netherlands [8] and Britain [9] have found substantially lower protection. The present contribution [10] is a welcome addition to the data base that can be used to address this important question. But much more remains to be done to standardize and improve methods, and to reconcile the results obtained from different approaches.

The impact of influenza is difficult to measure, because there is a great deal of influenza-like illness (ILI) in the world, caused by a large number of viruses, and only a very small percentage of cases is confirmed virologically [11]. Deaths triggered by influenza may be attributed to a number of final causes, including pneumonia, heart disease and stroke, and may occur weeks after initial infection [1,12,13].

The work of [10] is based on a long tradition of estimating influenza deaths by inference from seasonal patterns in death series. This method was pioneered by Serfling [14], and further developed by workers including Simonsen and colleagues [7,15]. While this work is valuable, and has produced apparently robust results, it is largely disconnected from quantitative virologic data about influenza. Thus, attribution of health effects to influenza is based strongly on assumptions about underlying death trends.

Recently, Thompson and colleagues have used virologic surveillance data to estimate influenza mortality [13] and hospitalizations [16], using a weekly, seasonal regression model. These models are the first to link quantitative virologic information to measures of influenza burden at the population level. The estimates produced are consistent with those of Serfling-like estimates [7,17] and are robust to the addition of estimates of respiratory syncytial virus (RSV) prevalence to the regressions. Questions remain about these estimates, however. The work of Thompson and colleagues removes a sinusoidal trend (fit at the same time as influenza prevalence), but does not take into account issues of autocorrelations; or the possibility of seasonal confounding between influenza prevalence, morbidity and mortality, and such factors as day length, temperature or school terms; or the likelihood that deaths caused by influenza infection in a given week may not occur until several weeks later.

Keatinge, Donaldson and colleagues [2,18], also used simple regression methods that ignored autocorrelations and seasonal confounding to study the causes of winter mortality in Europe. Unlike Thompson and colleagues, they lacked virological data and instead used proxies for influenza, but included temperature data, and found that temperature rather than influenza explained most of the excess deaths in their models.

Approaching a consensus on the health and mortality burden of influenza, and on the cause of winter excess mortality in general, is an important scientific and public-policy goal. For this to happen, further progess is needed in several areas.

• *Employing virological data*. When possible, analyses of influenza burden should be tied to estimates of laboratory-confirmed influenza cases. In some cases, such measures can be combined with ILI surveillance to improve estimates. Efforts should be made to increase the amount of viral surveillance information available in the public domain, with spatial and temporal break downs.

• *More sophisticated statistical analyses*. Time series methods that address issues of seasonal confounding and autocorrelation are available [19,20], but have been little used in analyses of seasonal mortality. Helfenstein [21] analyzed paired pre-whitened mortality series and inferred a "hidden relation" underlying heart disease in women and men. Much more needs to be done to investigate the relationship between mortality (or morbidity) and co-factors including weather, air pollution and epidemics of influenza and other viruses, while accounting for seasonal confounding and autocorrelations. Methods should evaluate multiple risk factors and consider the possibility of interactions between them.

• *Discuss and define time scales*. An important, and usually unasked, question in comparing results from different estimation approaches is the time scale on which influenza deaths are being measured. Everybody dies, so what is being measured as the mortality burden of influenza (or of weather) is deaths that are *hastened *by the cause in question. The question is whether these deaths are being hastened by weeks, months or years. Regressions that use a weekly time frame are expected to count deaths hastened by even a few weeks, while traditional methods of summing excess deaths over a season of 3 months or longer will be measuring at a different time scale.

• *Quantitative spatial comparisons*. As regional surveillance data, and data from different countries, become more available, analyses that explicitly incorporate risk factors and health outcome variables from various localities have the potential to greatly increase statistical power and shed light on unravelling the contribution of influenza and other risk factors to mortality and morbidity.

The contribution of influenza to morbidity and mortality – and, more broadly, cataloging the causes of daily and seasonal excess deaths and hospitalizations – remain as unresolved questions with important scientific and public-health implications. There is a pressing need for more communication between researchers studying different causes, places and time scales, and for application of appropriate, powerful statistical methods to these questions.

## Competing interests

The author(s) declare that they have no competing interests.
